# To the Editors of the Medical and Physical Journal

**Published:** 1803-11-01

**Authors:** H. E. Holder

**Affiliations:** Barbadoes


### To the Editors of the Medical and Physical Journal.

GENTLEMEN,

I Understand that in one of your late numbers, you have noticed the introduction of the Cow-pox into this island; if any credit is to be derived from it, I believe I may claim it. In July, 1801, I procured the vaccine matter in Liverpool; and on my arrival here in September following, inoculated nine girls at a parish school, in one of whom only the pustule was produced, and from her I propagated the disease. I have kept no regular account of my number of patients, but may safely affirm that Mr. Caddele, a surgeon, who afterwards inoculated in conjunction with me, and myself, produced the cow-pox in more than 1000 persons, blacks and whites; in no one case were any more pustules produced, than incisions were made. In some, after the disease had run its course, trifling eruptive complaints seemed to be excited by it, and in a few robust children, elevated itchy whales about the limbs and body accompanied the formation of the pustule, but a slight medical treatment removed these; in no other respect did the disease vary from its usual progress. As I was the original introducer of the practice here, (and I believe first succeeded in producing the disease in the West-Indies) the novelty of one disease preventing another for life, excited many doubts of its efficacy; and notwithstanding the inoculation was encouraged by the most respectable inhabitants, and every exertion was made by myself, Mr. Caddele, and I believe other practitioners, the island has been now destitute of matter for some months, from the want of subjects to re-produce it. I trust when next we procure a supply, to find less difficulty in diffusing its benefits.

I am, &c.

*Barbadoes, June* 10, 1803.

H. E. HOLDER, M. D.

P. S. I brought the vaccine matter from Europe between plates of glass, defended from the access of air by strips of bladder pasted round the edges. On thread, the heat of the climate appeared quickly to render it inert.

